# Supplementary material for: Analysis of S-Adenosylmethionine and S-Adenosylhomocysteine: Method Optimisation and Profiling in Healthy Adults upon Short-Term Dietary Intervention
Source: Metabolites. 2022 Apr 20;12(5):373. doi: 10.3390/metabo12050373 (PMC9143066; doi:10.3390/metabo12050373)
Supplement: Supplementary file 1 [file metabolites-12-00373-s001.zip › Supplementary Information Corrillero et al_28MAR2022.pdf]

# Supplementary Information for:

# Targeted analysis of S-adenosylmethionine and S-adenosylhomocysteine: method optimization and profiling in healthy adults upon short-term dietary intervention

Aida Corrillero Bravo[1†], Maria Nieves Ligero Aguillera[1†], Nahuel Rios Marzialli[1†], Lennart Moritz[1], Victoria Wingert[1], Katharina Klotz[1], Anke Schumann[1,2], Sarah C. Grünert[2], Ute Spiekerkoetter[2], Urs Berger[1], Ann-Kathrin Lederer[3], Roman Huber[3], and Luciana Hannibal[1]*

[1] Laboratory of Clinical Biochemistry and Metabolism, Department of General Pediatrics, Adolescent Medicine and Neonatology, Faculty of Medicine, Medical Center, University of Freiburg, 79106 Freiburg, Germany; aidacorrillero.ac28@gmail.com; nievesligero98@gmail.com; nahuelriosm@gmail.com; lennart.moritz@uniklinik-freiburg.de; wingertvictoria@gmail.com; katharina.klotz@uniklinik-freiburg.de; anke.schumann@uniklinik-freiburg.de; urs.berger@uniklinik-freiburg.de; luciana.hannibal@uniklinik-freiburg.de.

[2] Department of General Pediatrics, Adolescent Medicine and Neonatology, Faculty of Medicine, Medical Center, University of Freiburg, 79106 Freiburg, Germany; anke.schumann@uniklinik-freiburg.de; sarah.gruenert@uniklinik-freiburg.de; ute.spiekerkoetter@uniklinik-freiburg.de

[3] Center for Complementary Medicine, Department of Medicine II, Medical Center-University of Freiburg, Faculty of Medicine, University of Freiburg, 79106 Freiburg, Germany; ann-kathrin.lederer@uniklinik-freiburg.de; roman.huber@uniklinik-freiburg.de

†These authors contributed equally to this work
*Correspondence: luciana.hannibal@uniklinik-freiburg.de; Tel.: +49-761-270-43710

**Figure and Table legends in Supplementary Information**

**Figure S1.** Response of analyte and internal standard peak areas with varying injection volumes. Panel (a) SAM and $D_3$-SAM. Panel (b) SAH and $^{13}C_5$-SAH. Panel (c) Creatinine and $D_3$-creatinine.

**Figure S2.** Calibration curves for SAM and SAH over an expanded dynamic range (0-10 µM). Panel (a). Calibration curve of SAM (normalized with $D_3$-SAM as internal standard). Panel (b). Calibration curve of SAH (normalized with $^{13}C_5$-SAH as internal standard).

**Figure S3.** Age distribution of the adult healthy control group utilized to determine reference ranges for SAM and SAH.

**Figure S4.** Reported concentrations of plasma SAM. SAH and SAM:SAH ratios in published studies and in this work (Data from Table 5).

**Figure S5.** Correlation analysis of plasmatic and urinary SAM. SAH and SAM/SAH without the exclusion of outliers. Retaining the outliers skews the analysis significantly, as expected.

**Table S1.** Model selection for multiple linear regression analysis of metabolites in plasma and urine. Data used **without outliers**.

**Table S2.** Model selection for multiple linear regression analysis of metabolites in plasma and urine. Data used **with outliers**.

**Table S3.** Results of the multiple linear regression analysis of SAM. SAH and SAM/SAH in plasma and urine **with outliers**.

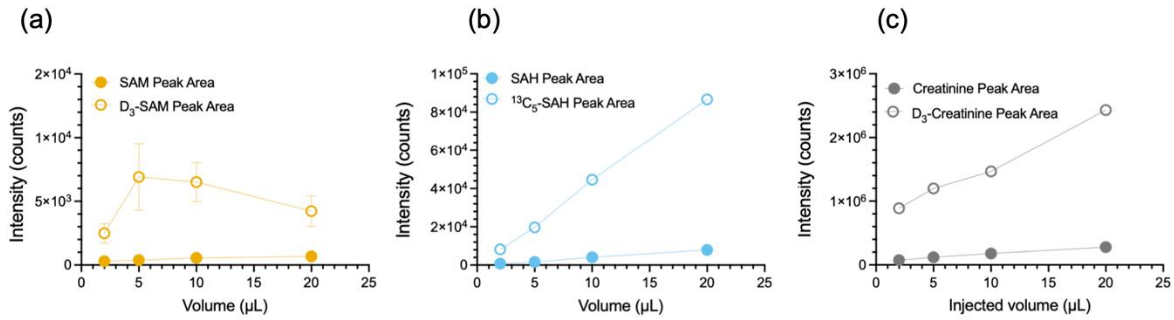


**Figure S1.** Response of analyte and internal standard peak areas with varying injection volumes. Panel (a) SAM and $D_3$-SAM. Panel (b) SAH and $^{13}C_5$-SAH. Panel (c) Creatinine and $D_3$-creatinine.

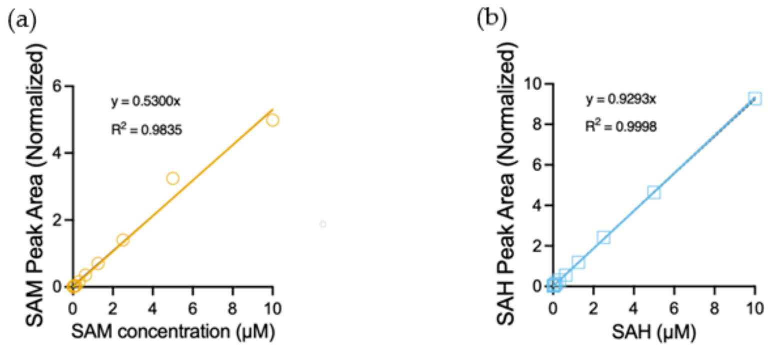


**Figure S2.** Calibration curves for SAM and SAH over an expanded dynamic range (0-10 µM). Panel (a). Calibration curve of SAM (normalized with $D_3$-SAM as internal standard). Panel (b). Calibration curve of SAH (normalized with $^{13}C_5$-SAH as internal standard).

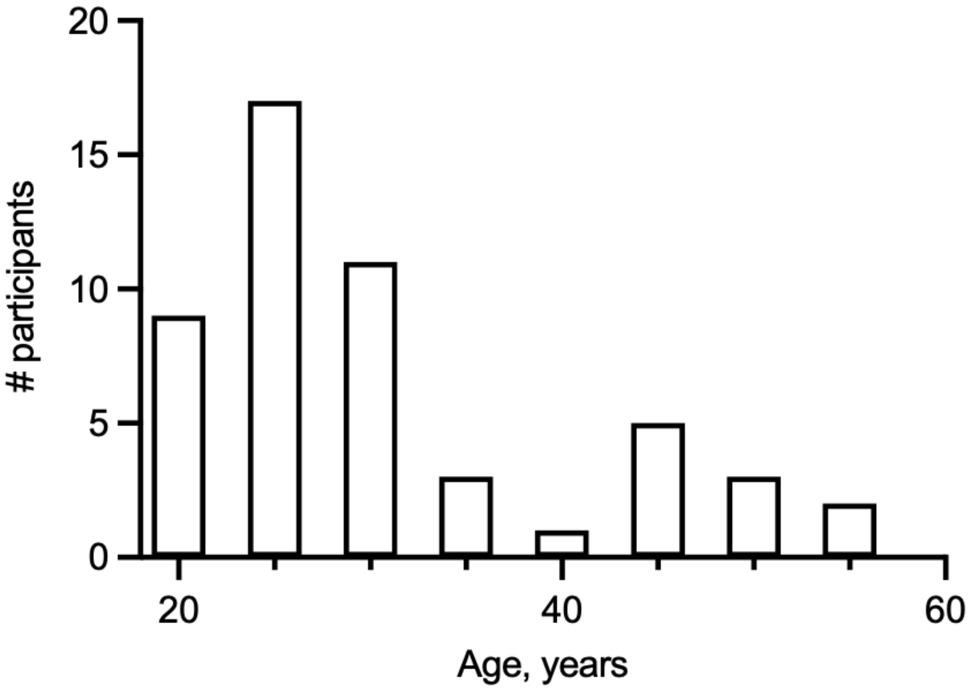


**Figure S3.** Age distribution of the adult healthy control group utilized to determine reference ranges for SAM and SAH.

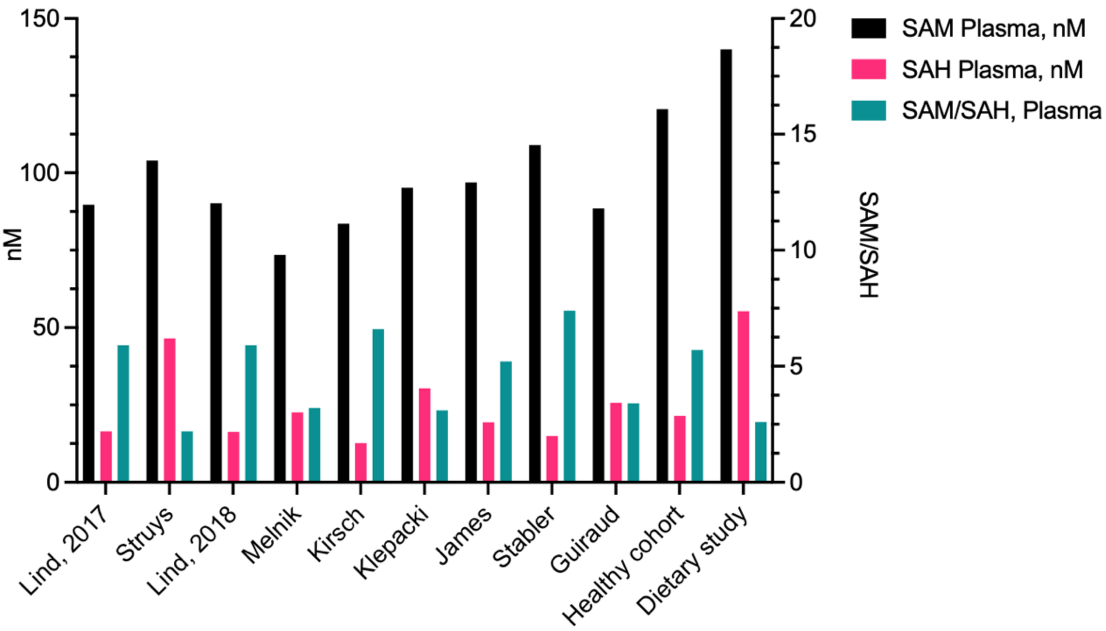


**Figure S4.** Reported concentrations of plasma SAM. SAH and SAM:SAH ratios in published studies and in this work (Data from Table 5).

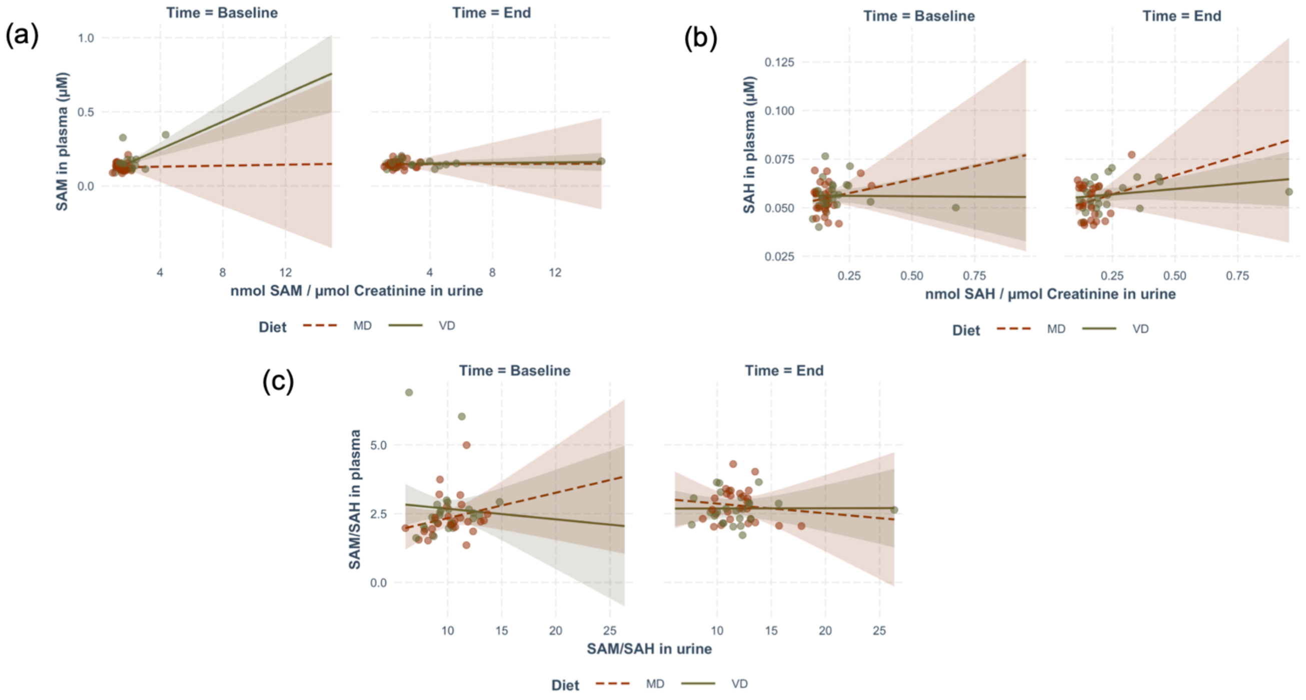


**Figure S5.** Multiple linear regression analysis of plasmatic and urinary SAM. SAH and SAM/SAH without the exclusion of outliers. Panel (a): SAM. Panel (b): SAH. Panel (c): SAM/SAH. Retaining the outliers skews the analysis significantly, as expected.

| **Table S1.** Model selection for multiple linear regression analysis of metabolites in plasma and urine. Data used **without outliers**. | | | | | | |
|---|---|---|---|---|---|---|
| | **SAM** | | **SAH** | | **SAM/SAH** | |
| **Interaction model** | **AIC** | **BIC** | **AIC** | **BIC** | **AIC** | **BIC** |
| Time x Diet | -446.63 | -431.06 | -661.06 | -645.55 | 163 | 178.51 |
| Metabolite x Diet | -446.55 | -430.98 | -661.52 | -646.01 | 163.54 | 179.05 |
| Metabolite x Time | -447.18 | -431.61 | -661.25 | -645.74 | 161.79 | 177.3 |
| Metabolite x Time x Diet | -441.37 | -418.02 | -658.46 | -635.19 | 165.85 | 189.11 |

| **Table S2.** Model selection for multiple linear regression analysis of metabolites in plasma and urine. Data used **with outliers**. | | | | | | |
|---|---|---|---|---|---|---|
| | **SAM** | | **SAH** | | **SAM/SAH** | |
| **Interaction model** | **AIC** | **BIC** | **AIC** | **BIC** | **AIC** | **BIC** |
| Time x Diet | -377.95 | -362.2 | -684.43 | -668.68 | 256.62 | 272.37 |
| Metabolite x Diet | -375.77 | -360.02 | -685.24 | -669.49 | 257.54 | 273.29 |
| Metabolite x Time | -392.73 | -376.98 | -684.6 | -668.85 | 258.24 | 273.99 |
| Metabolite x Time x Diet | -391 | -367.38 | -680.17 | -656.55 | 261.03 | 284.66 |

| **Table S3.** Results of the multiple linear regression analysis of SAM. SAH and SAM/SAH in plasma and urine **with outliers**. | | | | | | |
|---|---|---|---|---|---|---|
| | **SAM in Plasma** | | **SAH in Plasma** | | **SAM/SAH in Plasma** | |
| | **F-value** | **P-value** | **F-value** | **P-value** | **F-value** | **P-value** |
| Metabolite_Urine | 2.0958 | 0.151 | 1.8397 | 0.1782 | 0.0088 | 0.9256 |
| Time | 1.4635 | 0.2294 | 0.2073 | 0.65 | 1.3716 | 0.2445 |
| Diet | 1.1862 | 0.2789 | 0.8767 | 0.3515 | 0.4468 | 0.5055 |
| Metabolite_Urine x Time | 16.478 | **0.0001** | 0.5111 | 0.4765 | 0.1406 | 0.7085 |
| Metabolite_Urine x Diet | 0.8301 | 0.3646 | 1.3346 | 0.2509 | 0.1265 | 0.7229 |
| Time x Diet | 0.7954 | 0.3747 | 0.1655 | 0.685 | 1.1731 | 0.2815 |
| Metabolite_Urine x Time x Diet | 2.7484 | 0.1007 | 0.000 | 0.9967 | 1.1149 | 0.2937 |
